# Further Evidence on Online Video-Feedback Intervention: Enhancing Parent–Child Interactions and Reducing Disordered Eating in Children

**DOI:** 10.3390/ijerph21091260

**Published:** 2024-09-23

**Authors:** Silvia Cimino, Luca Cerniglia

**Affiliations:** 1 Department of Dynamic, Clinical and Health Psychology, Sapienza University of Rome, 00186 Rome, Italy; silvia.cimino@uniroma1.it; 2 Faculty of Psychology, International Telematic University Uninettuno, 00186 Rome, Italy

**Keywords:** online intervention, parent–child interactions, disordered eating, video feedback, maternal psychopathology

## Abstract

Background: This study evaluated the effectiveness of an online intervention designed to improve parent–child interactions and address disordered eating behaviors in children. Using remote video-feedback sessions based on observations of mother–child feeding interactions, the intervention offers a cost-effective and environmentally friendly alternative to traditional in-person methods. Aims: The study aimed to assess the impact of online intervention on the quality of mother–child interactions during feeding and its effectiveness in reducing psychopathological symptoms in both mothers and children. Methods: The intervention was conducted entirely online, with improvements measured using SVIA scores for mother–child interactions and the SCL-90/R Global Severity Index (GSI) for maternal psychopathological symptoms, alongside evaluations of children’s emotional and behavioral functioning, particularly anxiety, depression, and aggression. Results: The intervention significantly improved the quality of mother–child interactions across all SVIA subscales and led to reductions in maternal symptoms of depression and anxiety, as well as decreases in children’s emotional and behavioral symptoms. Conclusions: These findings suggest that online video-feedback interventions can effectively enhance mother–child relationships and reduce psychopathological symptoms in both mothers and children, although further research with larger sample sizes and more robust statistical analyses is needed to confirm these results.

## 1. Introduction

Eating difficulties are a significant concern during childhood, manifesting in two primary forms: clinically recognized eating disorders and subclinical or sub-threshold symptoms that may not meet the full criteria for a diagnosis but still pose significant challenges [1]. Research indicates that subclinical eating difficulties are widespread, with approximately one out of four of typically developing children experiencing such issues [2]. However, this prevalence dramatically increases to around eight out of ten in children who have developmental challenges, highlighting the heightened vulnerability in this group [3]. These findings underscore the importance of recognizing and addressing eating difficulties early, particularly in children with developmental concerns. The Developmental Psychopathology framework provides a comprehensive approach to understanding the factors that contribute to eating difficulties in children. Family environment plays a particularly crucial role in this interaction, as negative or maladaptive experiences within the family, such as exposure to parental psychopathology, can significantly increase the onset of disordered eating [4]. The quality of dyadic interactions is crucial in the development and maintenance of eating difficulties. Studies show that poor-quality interactions, particularly during feeding and play, are common in children with eating difficulties [5]. These interactions often involve either overly intrusive or withdrawn behaviors from the caregivers, which can disrupt the child’s ability to develop a healthy relationship with food [6]. However, protective factors can mitigate the risk of developing problems in the area of eating. Parental sensitivity to a child’s needs, particularly in recognizing and responding appropriately to hunger and satiety cues during nursing and weaning, is one of the most effective protective factors against the development of disordered eating behaviors [7]. Ensuring that parents are equipped with the knowledge and skills to foster these healthy interactions from an early age is essential for preventing the onset of eating difficulties.

Especially during and after the COVID-19 pandemic, traditional in-person assessments and interventions became challenging due to the need for social distancing and other public health measures. The possibility of another pandemic outbreak is not unlikely [8], and in general, sustainability and affordability of psychological interventions are key issues to take into consideration to grant inclusive possibilities of treatment to everyone. Traditional methods of parental education and support are often limited by geographical, financial, and logistical barriers, which can prevent families from accessing the help they need [9]. Digital platforms, however, offer a scalable solution that can reach a broader demographic, providing valuable resources to families regardless of their location or socio-economic status. These platforms can deliver evidence-based interventions that help parents understand and respond appropriately to their children’s needs, thereby promoting healthier developmental outcomes.

In March 2022, a study titled “A Study on Online Intervention for Early Childhood Eating Disorders during COVID-19” [10] was published in this journal. It presented the exploratory results of research entirely conducted online that sought to determine the effectiveness of digital tools in promoting positive parenting practices and reducing the risk of negative outcomes in children. Conducted over two assessment points, the study provided a longitudinal perspective on the intervention’s impact. Despite being a preliminary study with limitations such as a relatively small sample size and the lack of control groups, the findings were still noteworthy. The study focused on the quality of mother–child interactions during feeding, highlighting the importance of responsive and sensitive feeding practices for establishing a healthy foundation for a child’s behavioral and emotional regulation. The findings suggested that digital interventions could effectively support parents in maintaining these positive interactions, even in stressful situations.

Additionally, the study emphasized the critical role of maternal psychological well-being in the success of parenting interventions. Since maternal mental health is closely linked to parenting quality, addressing both aspects in interventions is likely to yield more positive outcomes for children [11]. This dual approach equips parents to provide better emotional support and foster healthy behavioral patterns in their children.

The present study intended to contribute to corroborate the method and procedure of the above intervention on the general population, as better explained in the Methods Section.

## 2. Materials and Methods

### 2.1. Participants

Two-hundred and sixteen mothers and their children were recruited between September and August 2023 in the general population through advertisements on online psychology research platforms and social media. The study employed a convenience sampling method, which is commonly used in research to obtain participants who are readily accessible and meet the study criteria [12]. Prior to participation, psychologists provided detailed information about the study’s objectives to the mothers. Those who expressed interest in participating signed a written informed consent form. The study was conducted in compliance with the Declaration of Helsinki and received approval from the Ethics Committee of the Psychology Faculty at Sapienza University of Rome (Protocol Number 0000809). The inclusion criteria for the study were infants aged 24 months, with difficulties in the area of feeding (undereating reported by the mothers) but no other physical or psychological problem. Families were excluded from the study if the children or mothers were receiving pharmacological or psychiatric interventions (N = 8), if the children had other physical or psychological problems (N = 4), or if the parents had reported existing or prior psychological conditions (N = 11). Additionally, families were excluded if the mothers did not complete all required questionnaires (N = 13) or did not complete all measures (N = 5). After applying these exclusion criteria, the final sample consisted of 175 families, with children aged 24 months (83 males and 92 females) and a mean age of 24.3 months (SD = 1.34). The mothers had a mean age of 35.02 years (SD = 2.45). The majority of participants were Caucasian (92%), with a significant portion of mothers having completed either high school or university education (88%). Most mothers were married (94%), and nearly all households were of average socioeconomic status (95% with an annual income of EUR 25,000–30,000). Additionally, all participating mothers reported high levels of satisfaction and comfort with telehealth and information and communication technologies (ICTs), as assessed by an adapted version of the Telehealth Satisfaction Scale [13].

### 2.2. Procedure

As stated above, this study was part of a larger study that addressed dyads with children with eating disorders [10] with the same procedure and measures. In this case, the study concentrated on the general population, accumulating data on the effectiveness of such intervention as a sustainable remote clinical intervention strategy for addressing disordered eating in children. As in the previous study, which recruited subjects in the clinical population, this research also collected data at two time points: T1 (baseline) and T2 (after four weeks of intervention). The intervention was delivered remotely, with sessions held twice a week for one hour each.

At both T1 and T2, mothers completed the Symptom Checklist-90-Revised (SCL-90-R) [14]. They also completed the Child Behaviour Checklist (CBCL 1½–5), which assessed emotional and behavioral functioning in children [15]. In addition, mother–child feeding interactions were remotely video-recorded for 20 min. These interactions were coded using a validated method, the Scale for the Assessment of Parent-Infant Interaction (SVIA), which was developed for assessing feeding interactions in both general and clinical populations [16]. The coding was performed by two independent raters who had been trained in the use of the instrument, with a high inter-rater reliability (Cohen’s kappa = 0.88) performed on 40% of the videos. The reliability of the SVIA coding system has been well-documented, with agreement rates between 0.86 and 0.91 in previous studies.

The intervention model used for supporting the parent–child relationship was based on the SVIA video recordings and adhered to the principles of video-intervention therapy (VIT) as described by Achenbach et al. [17] for addressing disordered eating in children. The clinical intervention employed in this study was grounded in video-feedback therapy, as outlined by Lucarelli et al. [18]. This approach is typically brief, with significant improvements observed within a few weeks. It integrates aspects of psychodynamic mother–infant psychotherapy and interaction guidance, drawing inspiration from Selma Fraiberg’s foundational work [19]. While focusing on observing the dyadic relationship, close attention is given to the mother’s concerns, anxieties, and narratives about her child, particularly her mental representations and projective distortions, which are verbalized to reduce their detrimental impact on parent–child interactions. Portions of these sessions are recorded and reviewed with the therapist via a videoconferencing platform, aiming to empower the parent in their caregiving role by offering new perspectives on the child’s behavior and suggesting practical steps for improving the quality of interactions. Although the primary focus is on the mother–child interaction patterns, it has been found that this treatment also led to reductions in both maternal and child psychopathological symptoms, including decreases in maternal depression and anxiety, as well as in children’s internalizing and externalizing behaviors. Further details about this technique can be found in the seminal work of Cramer and Palacio-Espasa [20]. Observational measures such as the SVIA are considered the gold standard for identifying early indicators of eating disorders as they provide objective assessments that are less reliant on potentially biased parent reports [16].

### 2.3. Measures

The Child Behaviour Checklist (CBCL 1½–5) is a widely used tool in international research for assessing emotional and behavioral problems in children. It includes scales for internalizing problems (covering emotional reactivity, anxiety/depression, somatic complaints, and withdrawal) and externalizing problems (including attention problems and aggressive behavior) [15].

The Symptom Checklist-90-Revised (SCL-90-R) is a 90-item self-report questionnaire that assesses psychological distress in adults. It includes nine dimensions: somatization, obsessive-compulsivity, interpersonal sensitivity, depression, anxiety, hostility, phobic anxiety, paranoid ideation, and psychoticism, along with a Global Severity Index (GSI). The Italian version of the SCL-90-R has shown good reliability, with Cronbach’s alpha values between 0.70 and 0.96 [14].

The SVIA (Italian adaptation of the Feeding Scale) assesses the quality of interactions during feeding by coding video-recorded sessions. It has been validated for use with children aged 12–36 months and provides a detailed analysis of relational dynamics during feeding, focusing on behaviors and emotional states that are indicative of relational difficulties [16].

### 2.4. Statistical Analyses

Data from T1 and T2 were analyzed using repeated measures analysis of variance (ANOVA) to compare scores on all measures. The statistical significance was set at *p* < 0.05. Mean values and standard deviations (SDs) were reported for all variables. A power analysis was conducted based on Cohen’s guidelines [18], with an alpha level of 0.05 and a power of 0.859, yielding a large effect size (f^2^ = 0.48). All statistical analyses were performed using IBM SPSS Statistics software, Version 25.0.

## 3. Results

The repeated measures ANOVA revealed significant main effects of time (all *p* < 0.001) on all four SVIA subscales. Post hoc tests using Bonferroni correction indicated that the SVIA scores at T2 were significantly lower than those at T1 across all subscales, suggesting improvements in the quality of mother–child interactions during feeding after the intervention. The specific subscales that showed significant improvement included the mother’s affective state, interactive conflict, food refusal behavior, and dyad’s affective state. The effect sizes (η^2^) and average scores at T1 and T2 for each SVIA subscale are detailed in Table 1.

The results suggested that the online intervention was effective in improving maternal emotional state and mother–child feeding interactions, thereby reducing the risk of developing more severe disordered eating behaviors in children. The study supported the feasibility and effectiveness of remote interventions for addressing early signs of disordered eating and improving the quality of parent–child interactions.

The analysis of variance (ANOVA) conducted on the Symptom Checklist-90-Revised (SCL-90-R) subscales and Global Severity Index (GSI) scores for mothers across the two time points revealed a significant main effect of time (*p* < 0.001). The results indicated that GSI scores were significantly lower at T2 compared with T1, suggesting an overall reduction in psychological distress following the intervention. Notably, the most affected subscales included depression, anxiety, and obsessive-compulsion, where mothers initially reported high levels of symptoms. Table 2 presents the mean scores and η^2^ values for these subscales, highlighting the changes observed across the intervention period.

Mothers rated their children’s emotional and behavioral functioning as less maladaptive at T2 compared with T1, particularly in the anxious/depressed and aggressive behavior subscales. Additionally, significant reductions were observed in the overall internalizing subscale, indicating an improvement in children’s emotional and behavioral outcomes following the intervention. The mean scores and η^2^ values for these subscales are detailed in Table 3, illustrating the positive impact of the intervention on reducing maladaptive behaviors in children.

## 4. Discussion

This study provides important insights into the effectiveness of an online intervention designed to improve parent–child interactions and address disordered eating behaviors in children. It followed a study conducted with the same methodology (but on a clinical population) by the same research group in 2022. As in the previous study, the intervention focused on remote video-feedback sessions following observations of mother–child feeding interactions, presenting a promising method to reduce both the financial burden of public health programs and the environmental impact by limiting the need for in-person visits.

The study’s results showed that the online intervention significantly enhanced the quality of mother–child interactions during feeding sessions. Specifically, improvements were observed in the SVIA scores at the second assessment (T2) compared with the initial assessment (T1), with substantial progress across all four subscales: the mother’s affective state, interactive conflict, food refusal, and the dyad’s affective state. These findings were consistent with previous research on in-person video-feedback interventions, which have been shown to effectively address feeding difficulties [21].

Beyond the improvements in interaction quality, the study found that the online intervention also led to significant reductions in psychopathological symptoms in mothers. Notable decreases were observed in the SCL-90/R Global Severity Index (GSI) and specific subscales related to depression and anxiety. This aligned with existing research that has established a close relationship between maternal depression and anxiety and disordered eating in children, as well as maladaptive parent–child interactions [22]. 

In addition to maternal outcomes, the intervention positively impacted the children’s emotional and behavioral functioning. Significant decreases were noted in the anxious/depressed and aggressive behavior subscales, as well as in internalizing behaviors. These results supported the existing literature linking low-quality parent–child interactions with children’s diminished ability to manage distress and regulate their emotions and behaviors [23,24,25,26,27]. The observed improvements in the children’s symptoms following the intervention suggested that enhancing the quality of the mother–child relationship could be a crucial mechanism for reducing psychopathology in children.

The study acknowledges the need for further research to confirm these findings. Future studies should include larger samples to explore data more thoroughly and to establish a stronger evidence base for the efficacy of online interventions in addressing parent–child interaction quality and child psychopathology.

## 5. Conclusions

This study provides further evidence for the effectiveness of an online intervention aimed at improving the quality of parent–child interactions during feeding. The intervention led to significant improvements in both maternal and child psychopathological symptoms. The video-feedback intervention connected closely with the positive outcomes in mother–child interactions during feeding. By allowing parents to review recorded sessions with a therapist, the intervention helped address maternal concerns and reshape projective distortions, enhancing the quality of caregiving. This real-time feedback fostered more sensitive parenting, leading to improvements in maternal mental health (e.g., reduced anxiety and depression) and better emotional regulation in children. This holistic approach emphasized both the mother’s psychological well-being and the quality of interactions, contributing to healthier child development outcomes. The findings suggest that this approach could serve as a viable and sustainable method for addressing disordered eating in children, offering the dual benefits of reduced in-person contact and lower intervention costs, while also contributing to a reduction in the environmental footprint associated with healthcare delivery. Future research should aim to replicate these findings with larger samples to better assess the effectiveness of online versus in-person interventions.

## Figures and Tables

**Table 1:** Average scores and standard deviations of the SVIA subscales and general quality of mother–child feeding interactions.

	T1	T2	η^2^
	M (SD)	M (SD)	
Mother’s affective state	23.12 (2.31)	19.32 (1.73) **	0.69
Interactive conflict	21.23 (2.42)	18.18 (2.21) **	0.71
Food refusal behavior	14.24 (1.36)	10.35 (1.39) **	0.66
Dyad’s affective state	14.15 (1.73)	11.83 (1.74) **	0.73
General quality	49.56 (3.52) ^§^	45.62 (2.44) ^§^**	0.68

^§^ At T1 and T2 no dyads exceeded the SVIA clinical cutoff of >54. η^2^: eta-squared. ** *p* < 0.001.

**Table 2:** Maternal scores at SCL-90/R.

	T1	T2	
	M (SD)	M (SD)	η^2^
SOM	0.34 (0.51)	0.32 (0.41)	0.17
O-C	0.71 (0.43)	0.68 (0.33)	0.34
I-S	0.42 (0.53)	0.39 (0.25)	0.18
DEP	0.76 (0.89)	0.61 (0.63) **	0.73
ANX	0.68 (0.71)	0.41 (0.52) **	0.86
HOS	0.42 (0.33)	0.39 (0.62)	0.20
PHOB	0.28 (0.43)	0.35 (0.39)	0.24
PAR	0.51 (0.65)	0.48 (0.79)	0.32
PSY	0.35 (0.54)	0.39 (0.49)	0.19
GSI	0.79 (0.53) ^§^	0.59 (0.59) ^§^**	0.74

^§^ At T1 and T2, no mothers exceeded the clinical cutoff of >1 at the SCL-90/R Global Severity Index (GSI). Note: SOM: somatization; O-C: obsessive-compulsive; I-S: interpersonal sensitivity; DEP: depression; ANX: anxiety; HOS: hostility; PHOB: phobic anxiety; PAR: paranoid ideation; PSY: psychoticism. η^2^: eta-squared. ** *p* < 0.001.

**Table 3:** Means (standard deviation) of child’s CBCL subscales.

	T1	T2	η^2^
E-R	3.44 (2.29)	3.39 (1.49)	0.25
A-D	6.41 (1.82)	5.12 (1.34) **	0.71
S-C	4.54 (2.11)	4.43 (1.29)	0.13
WIT	5.68 (1.48)	3.66 (1.98)	0.75
A-P	4.25 (1.52)	4.29 (1.56)	0.24
A-B	23.35 (4.17)	18.43 (3.22) **	0.75
INT	23.42 (2.49)	21.33 (2.59) **	0.72
EXT	20.31 (2.87)	20.69 (1.55)	0.29
DP	12.12 (1.43)	11.03 (2.31) **	0.68

Note: E-R: emotionally reactive; A-D: anxious/depressed; S-C: somatic complaints; WIT: withdrawn; A-P: attention problems; A-B: aggressive behavior; INT: internalizing problems; EXT: externalizing problems; DP: dysregulation profile. η^2^: eta-squared. ** *p* < 0.001.

## Data Availability

The data presented in this study are available at reasonable request to the authors.
